# TiO_2_ Polarons in the Time Domain: Implications for Photocatalysis

**DOI:** 10.1021/acs.jpclett.1c03677

**Published:** 2022-01-11

**Authors:** Alex J. Tanner, Geoff Thornton

**Affiliations:** Department of Chemistry and London Centre for Nanotechnology, University College London, 20 Gordon Street, London WC1H 0AJ, United Kingdom

## Abstract

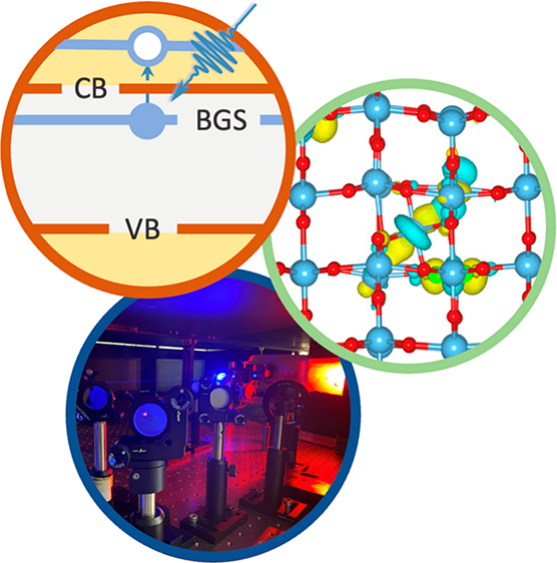

Exploiting the availability of solar energy to produce valuable chemicals is imperative in our quest for a sustainable energy cycle. TiO_2_ has emerged as an efficient photocatalyst, and as such its photochemistry has been studied extensively. It is well-known that polaronic defect states impact the activity of this chemistry. As such, understanding the fundamental excitation mechanisms deserves the attention of the scientific community. However, isolating the contribution of polarons to these processes has required increasingly creative experimental techniques and expensive theory. In this Perspective, we discuss recent advances in this field, with a particular focus on two-photon photoemission spectroscopy (2PPE) and density functional theory (DFT), and discuss the implications for photocatalysis.

Despite its band gap lying outside of the visible light spectrum, the stability and efficiency of TiO_2_ has led to its use as an industrial photocatalyst.^1−3^ Subsequently, the photocatalytic properties of TiO_2_ have also received widespread academic attention, resulting in an abundance of reviews^2−4^ and articles.^5−7^ Commercially, photocatalytic TiO_2_ is employed as a powder, commonly as the famed Degussa P25. This consists of a mixture of the two most abundant TiO_2_ polymorphs: anatase and rutile. Another feature of these powders is that they appear as such a brilliant white that they are used as a pigment, a result of the aforementioned wide band gap. However, surface scientists who study TiO_2_ at the atomic scale are familiar with TiO_2_ samples appearing blue. This blue hue arises from excess electrons in Ti 3*d* orbitals that are produced from chemical reduction, generally *via* the loss of O_2_ following sample preparation,^8−10^ or natural doping. It is well-known that these excess electrons exist as polarons,^11^ which can be thought of as a quasiparticle consisting of an electron surrounded by a virtual phonon cloud.

The behavior, size, and energy of electron polarons in the anatase and rutile phase of TiO_2_ are relatively well understood. A recent review by Franchini *et al.* covers these aspects excellently, and hence, this background will be discussed only briefly here.^12^ In both rutile and anatase TiO_2_ the Jahn–Teller splitting of Ti 3*d* atomic states in the pseudo-octahedral crystal field gives rise to orbitals of *t*_2*g*_- and *e*_*g*_-like symmetry. Polarons subsequently occupy a *t*_2*g*_-like state which is located below the Fermi level (*E*_F_). In the rutile phase, polarons form in an identical manner whether they arise from oxygen vacancies (O_vac_) (*via* the loss of O_2_) or doping. They localize as *small* (or Holstein) polarons.^13^ In this case, the surrounding ions screen the charge so that a potential well is formed.^14^ This results in a strongly bound electron species with a binding energy (BE) of ∼0.8–1.0 eV relative to *E*_F_, which can be observed in UV-photoemission spectroscopy (UPS).^15^ Rutile polarons have a low (∼95 meV) energy barrier for phonon-assisted hopping to adjacent Ti ions,^16^ which gives rise to conductivity that increases with temperature.^12^ The spin density of two polarons resulting from the formation of O_vac_ in the rutile phase of TiO_2_ is shown in Figure 1. Polarons in the anatase phase display more complex behavior. If excess electrons are introduced into stoichiometric regions of anatase TiO_2_ (*i.e.*, through doping, causing minimal lattice distortion) then polarons localize as *large* (or Fröhlich) polarons.^13,17^ These species are delocalized over several ions and have a BE of ∼40 meV.^13^ They display free-carrier-like properties which also give rise to conductivity in the sample, in this case decreasing with temperature. Polarons in anatase can also become trapped at O_vac_ sites, which can make up ∼15% of the surface region following ultrahigh vacuum (UHV) preparation.^18,19^ In this instance they exist as small polarons with a high energy barrier for hopping and hence remain trapped at the defect site.^13^

**Figure 1:**
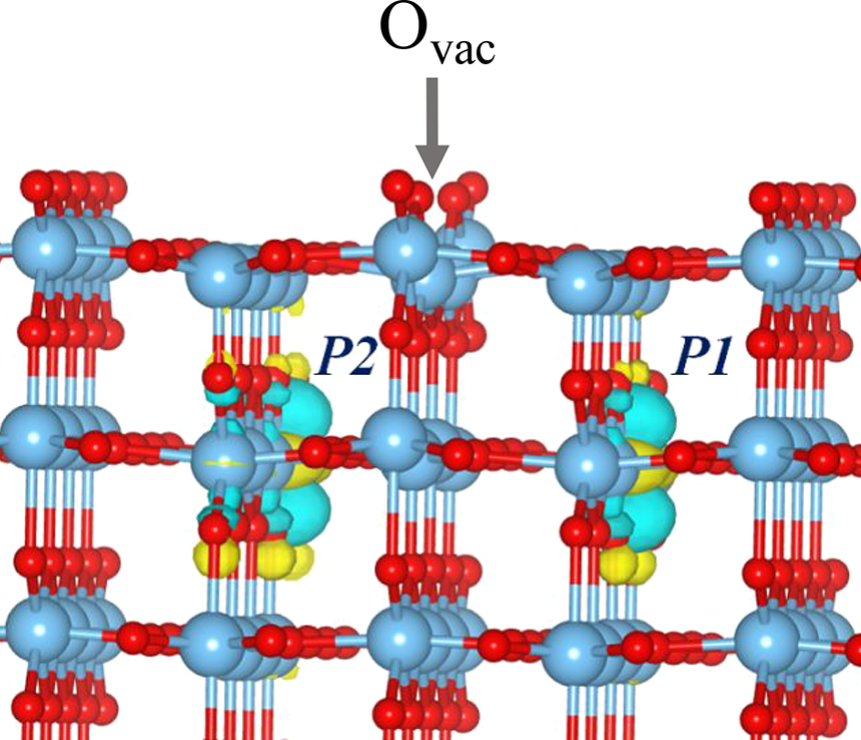
Spin density of two polarons (P1 and P2) in rutile TiO_2_(110) originating from the formation of O_vac_ (labeled). Red spheres represent oxygen (O) ions, and blue spheres represent titanium (Ti) ions. This model is a section of a 6 trilayer TiO_2_(110)-(4 × 2) slab. An isosurface of 0.05 (a.u.) was set to show the spin density contour of excess electrons. Blue and yellow contours come from different phases of wave function. Reproduced with permission from ref (40). Copyright 2021 American Physical Society.

What has been less clear is precisely how TiO_2_ electron polarons impact photocatalysis. Polaronic states can extend the light absorption of ground-state TiO_2_ into the visible^20,21^ and act as electron traps.^22^ However, while there is growing evidence that these states contribute positively to photocatalytic activity, it is not yet definitive. In fact, in a study by Wagstaffe *et al.* it was shown that polaronic states in anatase decreased the CO photooxidation rate. This contrasted with rutile, where the rate was found to increase.^23^ The conflicting behavior was attributed to the location of O_vac_, which are known to exist at the surface in rutile, but form in the subsurface in anatase. In another example, by Luttrell *et al.*, the photocatalytic degradation of methyl orange was found to increase when polaronic states were present in rutile TiO_2_.^7^ A rate increase was also evidenced by Zhuang *et al.* monitoring photodegradation of rhodamine B on rutile TiO_2_.^24^ These results have motivated further fundamental studies which have aimed to characterize the photoexcited transient behavior of polarons, predominantly through state-selective pump–probe spectroscopies and theoretical modeling. In recent years, these studies have added valuable information on polaron–light coupling, nonequilibrium dynamics, and the influence of adsorbates. In this Perspective, we provide our outlook on current developments, focusing on state-resolved studies of polarons in the time domain and the consequences for our understanding of photocatalysis.

Two-photon photoemission spectroscopy (2PPE) has emerged as a valuable tool for probing the excited transient states of polarons. Features in 2PPE spectra are most commonly produced as a result of coherent (simultaneous 2-photon excitation of an occupied state) or incoherent (two sequential 1-photon excitations *via* an intermediate state) processes (schematically shown in Figure 2a).^25^ This technique has distinct advantages over other pump–probe techniques in that it can resolve individual electronic states, which allows for greater engagement with theoretical calculations. In 2015, three articles emerged focusing on the photoexcitation of polarons in rutile TiO_2_(110), the most stable rutile facet. Although all groups observed a similar feature with an excited state centered around 2.6–2.8 eV above *E*_F_, the nature of the excited state character was interpreted differently.^26−28^ Because of the Jahn–Teller splitting of the 3*d* levels in the pseudo-octahedra crystal field, discrete unoccupied states of both *t*_2*g*_- and *e*_*g*_-like character arise. In principle, transitions from the *t*_2*g*_ ground state to either symmetry are possible. Recent DFT calculations provide strong evidence that the 2PPE feature of reduced rutile TiO_2_(110) is dominated by a *t*_2*g*_ → *t*_2*g*_ excitation feature,^29,30^ and from this point forward it will be referred to as such. However, it should be noted that there is not universal consensus on this assignment.^28,31^ A recent publication by Wang *et al.* discussed the merits of the computational methods used in each respective work.^32^ Another noteworthy aspect of rutile TiO_2_(110) 2PPE spectra is the influence of water. It is well-established that water dissociates at O_vac_ and forms bridging hydroxyls (OH_b_) at the TiO_2_(110) surface.^33^ Upon this reaction occurring, the *t*_2*g*_ → *t*_2*g*_ feature significantly increases in intensity but does not shift in energy.^26−28,34^ UPS and DFT suggest the reasons for this are multifaceted. Water not only draws subsurface polarons to the surface^15,29^ but also changes the initial state character to *d*_*xy*_, which couples more effectively with Ti^3+^ conduction band states.^29^ The tendency for polarons to occupy *d*_*xy*_ orbitals also has implications for the polarization-intensity dependence of the *t*_2*g*_ → *t*_2*g*_ feature, which is greatest when the electric field vector is perpendicular to the [001] azimuth. A characteristic 2PPE spectrum of hydroxylated rutile TiO_2_(110), taken from ref (27), is shown in Figure 2b.

**Figure 2:**
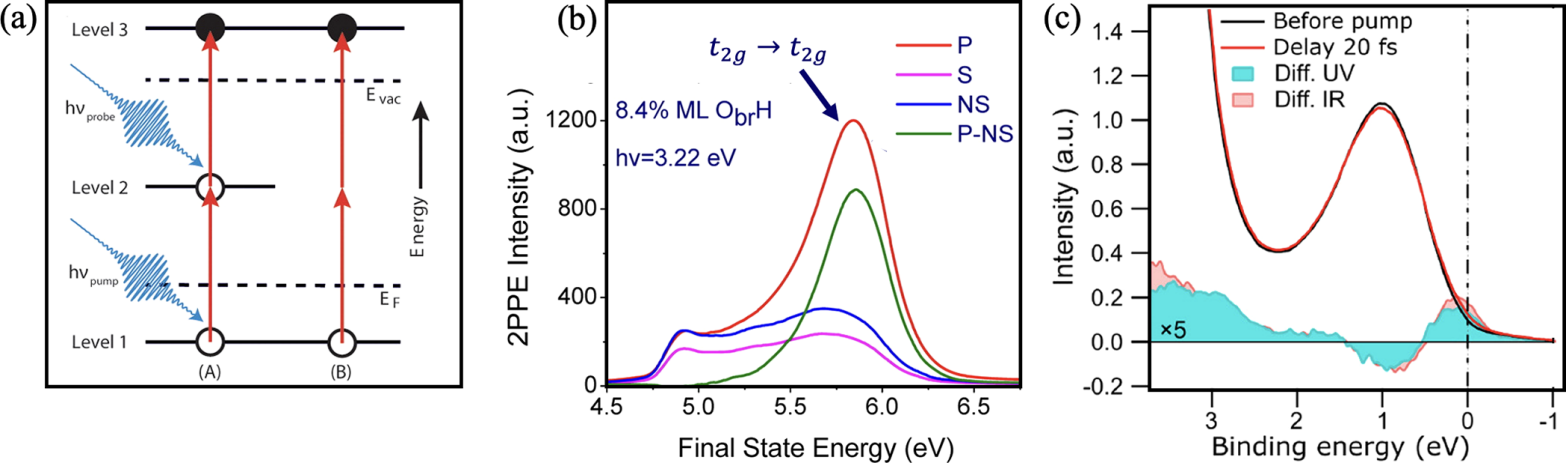
(a) Schematic of 2PPE excitation processes. 2PPE spectra can have two contributions, both originating from an occupied initial state (level 1) below the Fermi level (*E*_F_). Absorbing one photon allows stepwise, incoherent excitation (A) *via* an unoccupied, intermediate state (level 2) before a second photon probes the electron above the vacuum level (*E*_vac_) causing photoemission (level 3). Coherent excitation (B), where an electron at level 1 absorbs two photons simultaneously is also possible. Reproduced with permission from ref (26). Copyright 2015 American Chemical Society. (b) Typical 2PPE spectra for the hydroxylated rutile TiO_2_(110) surface taken with the [001] axis vertical. The spectra were measured with both p-polarized (P) and s-polarized (S) light with a photon energy of 3.22 eV. NS represents the s-polarized spectrum normalized to the secondary electron signal edge of the p-polarized spectrum. P-NS denotes the difference spectra of the p-polarized data minus the NS-polarized data. Energies are measured with respect to *E*_F_. Adapted with permission from ref (27). Copyright 2015 American Chemical Society. (c) Photoemission spectra (*h*ν = 30.4 eV) of the polaronic states before a UV pump pulse (black) and at a delay time of 20 fs (red). The difference between the two spectra is shown as the blue filled spectrum. As a comparison, the difference from an IR-pumped experiment (delay time 25 fs) is also shown by the filled red spectrum. Reproduced with permission from ref (35). Copyright 2019 American Chemical Society.

Zhang *et al.* used time-resolved (TR) UPS to study the lifetimes of photoexcited polarons in rutile TiO_2_(110).^35^ This technique allows for the depletion and generation of electron populations to be temporally profiled across the valence and conduction band. Fast recombination rates of 40–70 fs were reported for the direct retrapping of polarons. These are shorter than those of photoexcited electrons across the band gap (∼10 ps),^4^ which is likely due to differences in orbital character between the initial and excited state (Ti 3*d* → Ti 3*d* for polarons, compared to Ti 3*d* → O 2*p* for band gap recombination). A longer time scale component (ps) was also observed, which was assigned to the trapping of conduction band electrons, created by band gap excitation, as polarons. The time scales of direct polaron retrapping are possibly inhibitive to their role in photoinduced redox chemistry.^35^ However, another interesting observation was noted. With a 3.5 eV UV pump photon, coinciding with the resonant photon energy for polaron excitation, the spectra were dominated by electron-transfer processes to and from the polaronic states. This is shown in Figure 2c. It may be expected that the spectra would have been governed by band gap excitation. The explanation given for this was that at 3.5 eV the valence band density of states (DOS) accessed was not significantly higher than that of the polaronic states. However, DFT calculations by Wen *et al.* suggested an additional reason. They showed that compared to polaron photoexcitation, band gap excitation (from the valence band edge) displayed significantly lower oscillator strengths.^30^ The onset for the *t*_2*g*_ → *t*_2*g*_ excitation in the rutile phase is 3.1 eV,^26^ coinciding with band gap excitation. These results suggest that at band gap energies, polarons may contribute a greater abundance of photoexcited charge carriers than valence band states.

The 2PPE spectra of polarons in the anatase phase are more varied, with data for the stable (101) termination being reported in three publications.^30,36,37^ All point to a weak 2PPE resonance compared to rutile, which is now understood to be due to the subsurface location of O_vac_. In a publication by Payne *et al.* it was demonstrated that if surface O_vac_ are generated by an electron beam, the 2PPE yield increased significantly.^36^ Intriguingly, it has also been shown that anatase polarons couple strongly with a Ti^3+^ conduction band state 2.0 eV above *E*_F_, giving rise to a 2.8 eV resonant photoexcitation scheme.^37^ This is less than the anatase band gap of 3.2 eV, which is in line with reports that Ti^3+^ self-doping of anatase gives rise to an extended absorption spectrum.^20^ Calculations suggest that like rutile, the initial and excited state in this scheme are *t*_2*g*_ in character.^30^ In the anatase TiO_2_(101) case, polaron photoexcitation is enhanced when the electric field vector is perpendicular to the [010] azimuth.^30,37^

The polarization dependence of 2PPE intensity is proportional to the transition dipole moment (μ) from an initial state, |*i*⟩, to an intermediate state, |*j*⟩, and from |*j*⟩ to a final state, |*p*⟩, above the vacuum level.^38^ This proportionality is given by
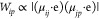
1where *W*_*ip*_ is the two-photon transition rate from |*i*⟩ to |*p*⟩ and e is the normalized electric field at the surface. The oscillator strength of the initial transition from |*i*⟩ to |*j*⟩ can be calculated through the following equation:^29,39^
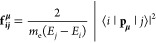
2where **f**_***ij***_^**μ**^ is the oscillator strength in the  polarization direction. ⟨*i*| and |*j*⟩ denote the Kohn–Sham orbitals corresponding to the initial state of the polarons and intermediate, respectively, and *E*_*i*_ and *E*_*j*_ are the corresponding eigenvalues. **p_*μ*_** is the momentum operator along . These calculations have allowed greater detail to be extracted from 2PPE measurements and, coupled with DOS calculations, have provided impressive insight. An important factor seems to be the size of the model used. Those calculations performed on six-layer slabs^29,40,41^ appear to match experiment closer than those performed on four-layer slabs.^27,30^ It should be noted that the sample probing depth in 2PPE experiments is typically greater than the slab thickness of the TiO_2_ models, potentially providing a limitation. The HSE06 functional has been increasingly used and has been shown to describe polaronic states with good accuracy. This has been particularly valuable as the experimental systems studied have increased in complexity.

One such example is our recent 2PPE and DFT study which determined that polarons in the bulk of rutile TiO_2_ can contribute to the 2PPE signal.^40^ Polarons in the bulk are less bound than at the surface of rutile TiO_2_ and undergo a 0.2 eV offset excitation channel with the same resonant photoexcitation energy. Characterizing the photoexcitation behavior of bulk polarons is valuable for photocatalysis as they are more abundant than surface polarons and are protected by the lattice from oxidation. More recent work by Wang *et al.* suggests that the difference between surface and bulk polaron excitation also contains an anisotropic component where polarons excited in the [110] direction are stabilized.^32^ Enticingly, these works (as well as previous work by Zhang *et al.*(26) and Mao *et al.*(42)) suggest that the interaction between light and polarons can be tuned depending on their local environment. This has become evident in 2PPE studies of more complex TiO_2_ surface environments. In our 2PPE and DFT study of formate and acetate overlayers on rutile TiO_2_(110), we demonstrated that electron polarons can couple with carboxylate adsorbates to change the local crystal field.^41^ For formate this gives rise to additional high oscillator strength transitions in the Ti^3+^ conduction band, specifically a *t*_2*g*_ → *e*_*g*_ transition where the excited state is centered 3.83 eV above *E*_F_. The results of oscillator strength calculations for the formate-saturated termination of rutile TiO_2_(110) are shown in Figure 3a. One reason this could impact photocatalysis is that these higher energy transitions may exhibit significantly different recombination times. Furthermore, polaronic states located close to the adsorbate will increase the probability of charge transfer, which is vital for photodegradation. The anatase-formate case is also intriguing. Formate adsorption causes subsurface O_vac_ to diffuse to the surface.^37,43^ Because polarons stay fixed at O_vac_ sites in anatase TiO_2_(101), they are particularly sensitive to the local environment. The initial occupied state shifts 0.3 eV higher in BE, which can be excited into states 3.0 eV above *E*_F_.^37^ Moreover, because these states are now located at the surface, the 2PPE yield is vastly increased (see Figure 3b). The capability to tune polaron–light resonances could be key in photocatalytic design, which gives rise to numerous potential avenues of investigation. For example, the coupling profile of rutile TiO_2_ polarons to CO has been shown to display a dependence on the sample reduction level.^44^ This interplay may be a way to engineer polaronic resonance states.

**Figure 3:**
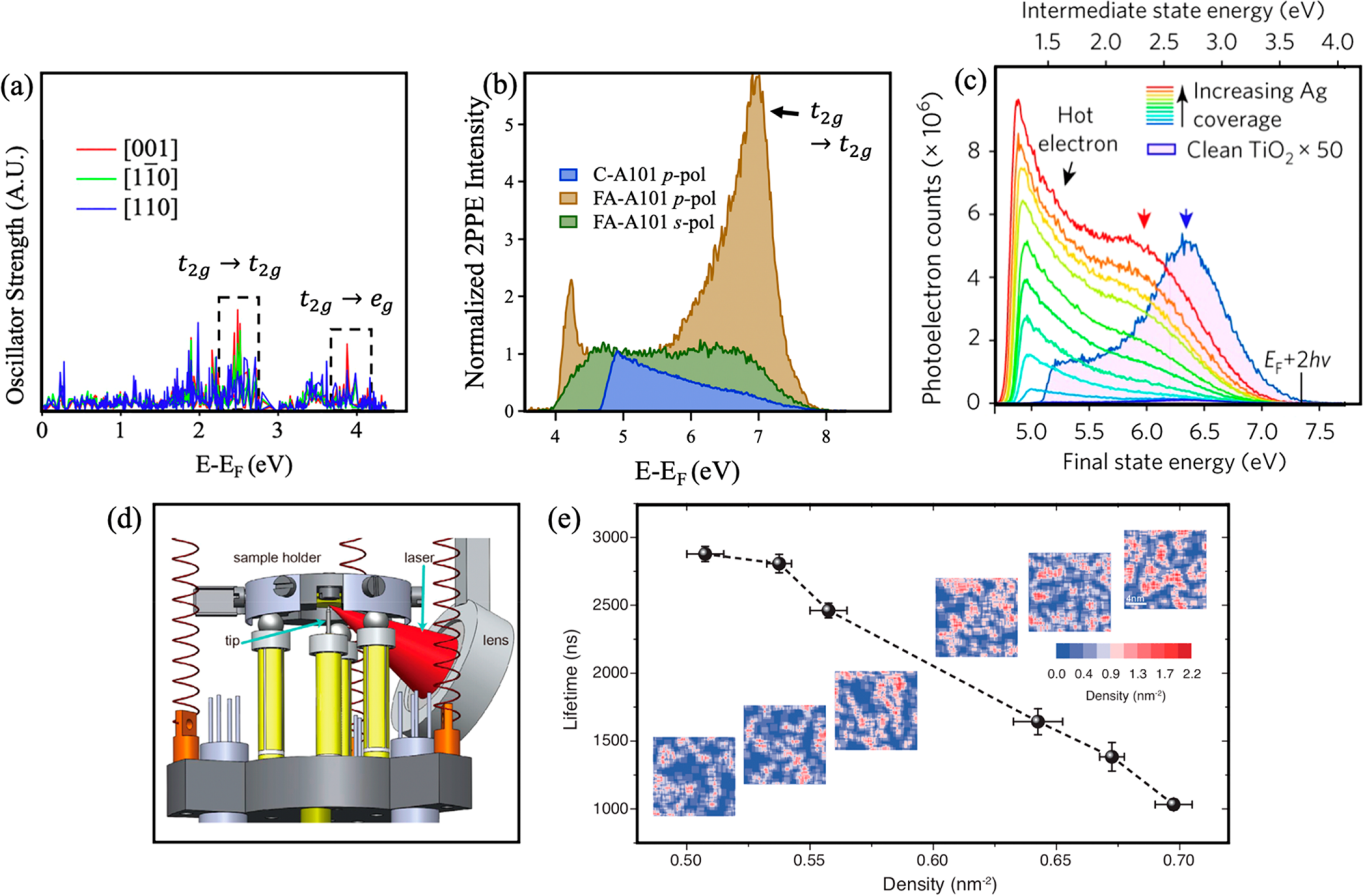
(a) Computed oscillator strengths for transitions from polarons to the conduction band on formate terminated rutile TiO_2_(110). Red [001], green [110], and blue [110] represent directions of transition dipole moments. Boxed peaks in the oscillator strengths coincide with *t*_2*g*_ → *t*_2*g*_ and *t*_2*g*_ → *e*_*g*_ excitations. Adapted with permission from ref (41). Copyright 2021 American Chemical Society. (b) 2PPE spectra of as-prepared clean anatase TiO_2_(110) (C-A101) and the formate terminated surface (FA-A101) (*hν* = 3.87 eV (320 nm)) normalized at 5.2 eV (*E* – *E*_F_). The polarization of light is shown in the panel legend, and the [010] azimuth is vertical. Adapted with permission from ref (37). Copyright 2021 American Chemical Society. (c) The p-polarized 2PPE spectra (*hν* = 3.65 eV) obtained with increasing Ag coverage on TiO_2_(110). Blue-shaded spectra (note the ×50 multiplication) are of the reduced TiO_2_ surface; the blue arrow marks the *t*_2*g*_ → *t*_2*g*_ transition peak (originally labeled *t*_2*g*_ → *e*_*g*_ in ref (50)). On deposition of Ag, the hot electron (black arrow) and the interface-state contributions (red arrow) exhibit a greatly enhanced 2PPE yield. Adapted with permission from ref (50). Copyright 2017 Springer Nature. (d) Zoomed-in schematic of the homemade beetle-type scanner for optical pump–probe experiments. A high-precision 3D nanopositioner controls a spheric lens near the STM tip. Reproduced from ref (51). Copyright 2020 American Physical Society. (e) The dependence of free electron lifetime on the O_vac_ defect density, extracted from STS data. The O_vac_ density was calculated within 20 × 20 nm^2^ areas. The error bars of the lifetime and the O_vac_ density arise from the fitting error and the statistical error, respectively. The insets are the defect density mapping of six 20 × 20 nm^2^ areas with the averaged defect density ranging from 0.51/nm^2^ to 0.70/nm^2^. The local density was analyzed in square areas of 1.8 × 1.8 nm^2^, which corresponds to the resolution of the density mapping. Reproduced from ref (51). Copyright 2020 American Physical Society.

The interactions of metal clusters with TiO_2_ are of great interest because of well established increases in photocatalytic activity.^45,46^ Whether polarons transfer to metal clusters appears to depend on the sample reduction level and cluster size.^47−49^ In a 2PPE study of Ag nanoparticles (NP) on rutile TiO_2_(110), Tan *et al.* reported the quenching of the *t*_2*g*_ → *t*_2*g*_ feature despite their calculations showing that charge transfer occurred to the substrate.^50^ They noted that at this interface, the 2PPE spectra were dominated by plasmonic modes and an “induced interface state”. The 2PPE spectra of rutile TiO_2_ with increasing Ag NP coverage are shown in Figure 3c. To our knowledge no other 2PPE studies of metal–TiO_2_ interfaces have been reported, and it is not clear how polarons will impact the photocatalytic properties of these systems. Further insights may be gained from studies of other substrate–metal combinations with a range of cluster sizes.

Although 2PPE can probe individual electronic states, its sampling area is in the macroscopic regime, meaning atomic precision is not possible. A recent study provided an exciting update by presenting the first demonstration of nonequilibrium polaron dynamics in TiO_2_ at the atomic scale.^51^ This was achieved by coupling a 5 K scanning tunneling microscope (STM) with a pulsed ns laser setup to perform time-resolved scanning tunneling spectroscopy (TR-STS) (see Figure 3d). The two key results from Guo *et al.* were obtained from photoexcitation in the steady state (quasicontinuous laser) and those measured under dynamic control (ns pump–probe laser). The steady-state results show that at 700 nm irradiation, polarons undergo transitions to conduction band states and their occupied state BE exhibits a downward shift. Subsequent calculations revealed this was due to a decrease in the on-site Coulomb interaction energy when polarons are removed from the in-gap state. In the dynamic measurements, the photoinduced tunnelling current (*I*_ph_) was measured versus the delay time (*t*_d_) of two 532 nm laser pulses with the bias set to the conduction band or valence band tail. The results suggest lifetimes of approximately 3.0–3.6 μs for photoexcited polarons. Lifetimes of this order have been noted in other work with ns lasers,^52^ although as noted above, much shorter lifetimes should also be detected with fs lasers. Guo *et al.* used the dynamic measurements to demonstrate that the excited-state lifetime decreased linearly with increasing oxygen vacancy density (see Figure 3e). This was attributed to the diffusion length of conduction band polarons, which is assumed to be shorter at higher defect densities.

Absorption spectroscopies have also been applied to characterize the transient behavior of excited polarons. IR spectroscopy provides an interesting comparison as photon energies can be used that do not promote polarons above the conduction band minimum. Sezen *et al.* found that polarons can undergo transitions within their potential well to photoexcited “hydrogenic” states, corresponding to sharp peaks in the IR spectrum (see Figure 4a).^53^ Furthermore, they compared their results from single crystals to those from powdered samples and found identical features in both cases, confirming the presence of polaronic states in the powder. In another example, Santomauro *et al.* used fs Ti *K*-edge X-ray absorption spectroscopy (XAS) to characterize the transient behavior of photogenerated polarons in colloidal anatase. In their work a 3.50 eV (355 nm) pulsed excitation source was used and a time resolution of approximately 200 fs was obtained.^54^ The progression of the Ti *K*-edge at 4.982 keV with increasing delay times is shown in Figure 4b, where the gray “fit” indicates that polaron formation following photoexcitation occurs <300 fs, which is a similar order to the values reported by Zhang *et al.* with TR-UPS.^35^

**Figure 4:**
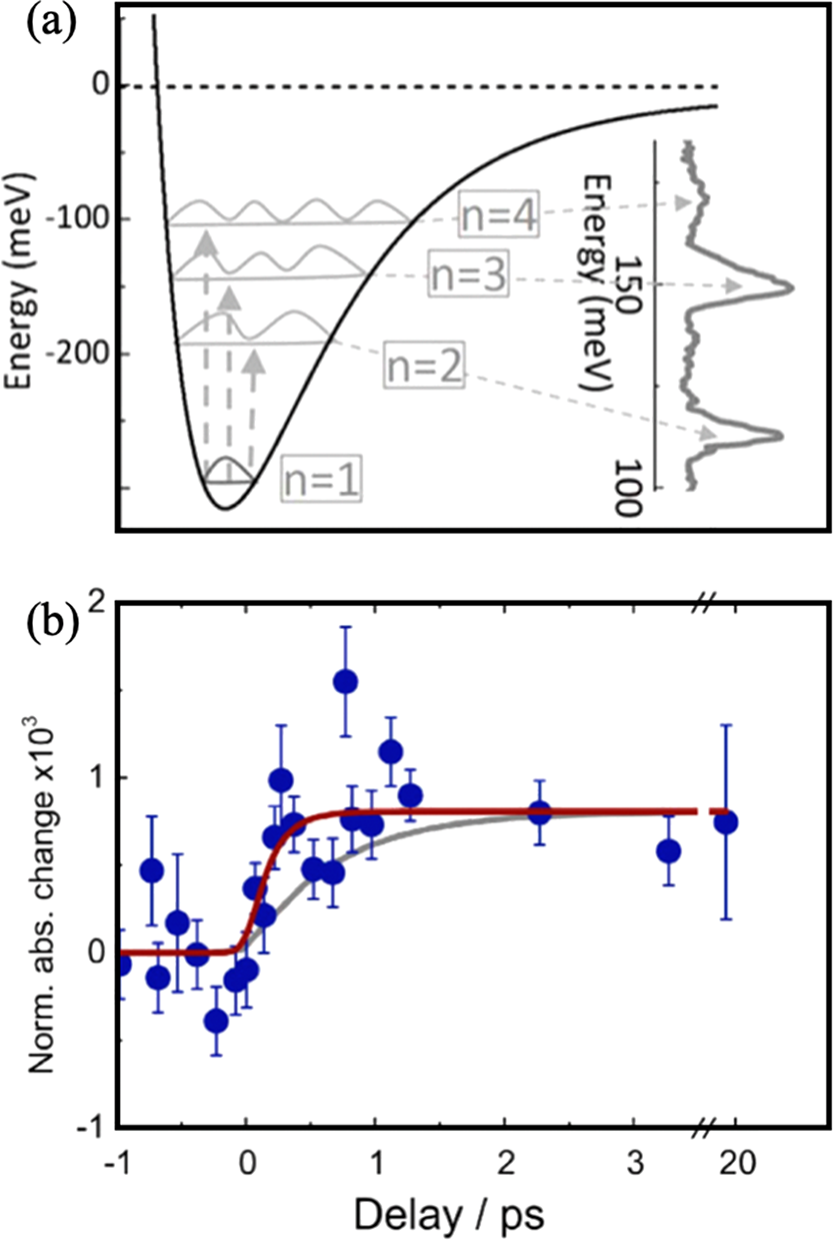
(a) Hydrogenic potential at a polaron Ti^3+^ site in bulk rutile showing the different excitations giving rise to absorption bands in the IR data. A polynomial baseline was subtracted from the raw IR data to enhance the visibility of additional hydrogenic states. Reproduced with permission from ref (53). Copyright 2014 Springer Nature. (b) Temporal evolution of the photoinduced X-ray absorption change at 4.982 keV of room-temperature colloidal TiO_2_ nanoparticles excited at 355 nm (blue dots). After the rise, the signal remains constant up to the limit of the time scan (50 ps). The brown trace represents the data fit, giving a rise time of 170 fs. The gray trace shows a satisfactory fit of the data with the longest rise time (300 fs), which is an upper limit. Reproduced with permission from ref (54). Copyright 2015 Springer Nature.

Activity studies have clearly established that polarons impact the photocatalytic reaction rate of TiO_2_, with most studies demonstrating that this influence is positive. State-selective pump–probe spectroscopies such as 2PPE, TR-UPS, and TR-STS, as well as state-of-the-art theory, can assist in understanding the symmetries, lifetimes, and energies that govern these effects. Indeed, numerous time- and state-resolved studies have added valuable information on the nonequilibrium processes of polarons, despite the challenge of their inherently short recombination rates. These recombination times do of course limit the efficiency of polarons in directly activating photochemistry. However, the ability to manipulate the energy and location of electron polarons gives rise to the potential that their dynamics, and thus activity, may also be tuned. This may especially be the case in anatase TiO_2_ where the low mobility of defect (O_vac_) polarons makes their energies easier to manipulate and may result in longer recombination rates after photoexcitation. Furthermore, there is evidence to suggest polaronic states can alter the lifetime of photoexcited band electrons, trapping them on the ps time scale. These species are significantly more likely to perform desirable redox chemistry. The extent that polarons impact band gap photoexcitation processes will be a key objective for future studies.

The clear question is how to build on our current understanding. 2PPE still has much to offer. Only recently have these experiments ventured into adsorbate structures and bulk materials. To our knowledge, only one 2PPE study of TiO_2_’s minority facets of either rutile or anatase exists,^32^ despite clear differences in the polaronic occupied energy. This information will be valuable as we build our picture of how surface structure influences polaron behavior. Two-color 2PPE (*hv*_pump_ ≠ *hv*_probe_) may also add understanding to this area, especially in the low-energy excitation regime. As evidenced in this Perspective, polarons can interact with light in the IR and visible regime. However, one-color 2PPE (*hv*_pump_ = *hv*_probe_) at these energies is almost impossible to study because of limitations associated with the magnitude of the sample work function. The increasing accessibility to X-ray free electron lasers (XFELs) gives rise to a host of exciting possibilities. This is evidenced by recent work that monitored the dynamics of CO oxidation on rutile TiO_2_(110).^55^ By tuning the pump photon energy to below that of the band gap it may also be possible to isolate polaron contributions to the photocatalytic yield of model TiO_2_ photochemical reactions.
